# MEKK3 bridges gut-brain communication and cerebral cavernous malformation pathogenesis

**DOI:** 10.1038/s41420-026-03062-6

**Published:** 2026-03-27

**Authors:** Peng Cheng, Hongkuan Han, Ying Huang, Yang Shen, Xuan Jiang, Xiaoxiong Song, Cheng Qian, Lei Chen, Yang Zhao

**Affiliations:** 1 Drum Tower Hospital Clinical College, School of Medicine, Nanjing University of Chinese Medicine, Nanjing, 210023 China; 2 School of Life Science and Technology, Key Laboratory of Developmental Genes and Human Disease, Southeast University, Nanjing, 210031 China; 3 Institute of Translational Medicine, School of Medicine, Zhejiang University, Hangzhou, 310029 China; 4 Jiangsu Key Laboratory for Pharmacology and Safety Research of Chinese Materia Medica, Nanjing University of Chinese Medicine, Nanjing, 210023 China; 5 Institute of Microphysiological Systems, Southeast University, Nanjing, 211189 China

**Keywords:** Mechanisms of disease, Biogeochemistry

## Abstract

Cerebral cavernous malformation (CCM) is a condition affecting the brain vasculature, characterized by endothelial dysfunction and abnormal vascular structure. In recent years, the gut-brain axis has emerged as a significant regulatory factor influencing cerebrovascular health. The gut microbiota, through its metabolites, immune modulation, and signaling interactions with the brain, plays a critical role in the pathogenesis of CCM. Research indicates that dysbiosis can trigger systemic inflammatory responses via pathways such as lipopolysaccharide (LPS) -TLR4, short-chain fatty acids (SCFAs), and trimethylamine N-oxide (TMAO), ultimately affecting cerebrovascular function and the integrity of the blood-brain barrier. Additionally, the gut-brain axis may influence the proliferation, migration, and apoptosis of endothelial cells, potentially promoting or inhibiting the development of CCM. Although the exact mechanisms linking the gut-brain axis and CCM remain unclear, existing studies suggest a potential key role in the pathological progression of CCM. This review explores the mechanisms by which the gut-brain axis contributes to CCM and proposes that targeting relevant pathways within the gut-brain axis may offer new therapeutic strategies for CCM.

## FACTS

- The gut–brain axis has been increasingly implicated in cerebrovascular disorders, yet how microbiota-derived metabolites influence endothelial signaling through MEKK3 remains largely unexplored.
- MEKK3–KLF2/4 signaling is a central determinant of vascular stability in CCM, but its upstream microbial and immune regulators have not been systematically defined.
- Specific microbial metabolites, including SCFAs, LPS, TMAO, and indole derivatives, exert dual effects on cerebrovascular integrity with both protective and deleterious properties, and the mechanistic basis underlying the differentiation of these opposing biological effects remains largely elusive.
- The immune-endothelial-microbiota axis, particularly via TLR4-MEKK3 crosstalk, represents a potential amplifier of neurovascular inflammation that could become a therapeutic target for CCM intervention.
- Whether modulating gut microbiota or intestinal barrier function can effectively attenuate MEKK3-driven vascular lesions in vivo remains a major question for future translational research.

## Introduction

Cerebral cavernous malformation (CCM) is a rare yet potentially devastating cerebrovascular disorder, with an estimated prevalence of approximately 0.1–0.5% of the population worldwide [1, 2]. Symptomatic cases of CCM occur at a rate of 0.2–1.9 per 100,000 people annually, with 20–50% of patients presenting with significant neurological symptoms, including seizures, brain hemorrhages, headaches, and cognitive dysfunction [3–5]. Advances in neuroimaging techniques have enhanced the detection of CCM, particularly among asymptomatic carriers, with many cases identified through magnetic resonance imaging (MRI), especially using gradient echo (GRE) or susceptibility-weighted imaging (SWI) [6]. However, the global incidence of CCM may still be underestimated, as asymptomatic cases may go undiagnosed throughout life [7, 8]. Given the potential risk of spontaneous cerebral hemorrhage, particularly in lesions situated in the brainstem or those with active leakage, it is imperative to investigate the underlying mechanisms of cerebral cavernous malformation and develop targeted therapeutic interventions.

The gut-brain axis (GBA) is a complex signaling network involving the nervous, immune, and endocrine systems, playing a critical role in maintaining neurovascular homeostasis and regulating brain function [9–11]. This axis includes neural pathways (such as the vagus nerve and enteric nervous system), immune responses, blood circulation, and metabolites derived from the gut microbiota, all of which influence brain health through various biological signaling mechanisms [12, 13]. Growing evidence increasingly suggests a strong association between CCM progression and the gut-brain axis, particularly through the influence of gut microbiota and its metabolites [14, 15]. These metabolites regulate brain inflammation and immune responses, potentially contributing to cerebrovascular damage through several mechanisms: (1) altering the permeability of the blood-brain barrier [16, 17], (2) modulating endothelial cell signaling pathways [18, 19], and (3) influencing neuroimmune networks. For instance, short-chain fatty acids (SCFAs), such as butyrate and acetate, modulate endothelial function via G protein-coupled receptors (GPR41/43), while indole metabolites and trimethylamine N-oxide (TMAO) may impact CCM progression via distinct signaling pathways [20–23]. Despite these insights, the precise mechanisms by which microbiota-derived metabolites affect CCM remain unclear and require further investigation.

In this review, we will explore the interactions between gut microbiota, gut microbiota-derived metabolites, and the progression of CCM, with a focus on their potential effects on the onset and development of the disease. We will propose future directions to offer new perspectives on the mechanisms of CCM and potential therapeutic strategies.

## Genetic and regional variations in the pathogenesis of CCM

CCM exhibits substantial regional, genetic, and clinical heterogeneity across populations [24]. Sporadic CCMs represent the predominant disease form, accounting for more than 80% of cases and typically manifesting as isolated vascular lesions [25, 26]. Although early studies primarily attributed sporadic CCM pathogenesis to endothelial injury, dysregulated inflammatory signaling, and altered hemodynamic forces [27–29], emerging genetic evidence indicates that somatic mutations play a decisive role in determining lesion growth, hemorrhagic potential, and clinical aggressiveness.

**Fig. 1:**
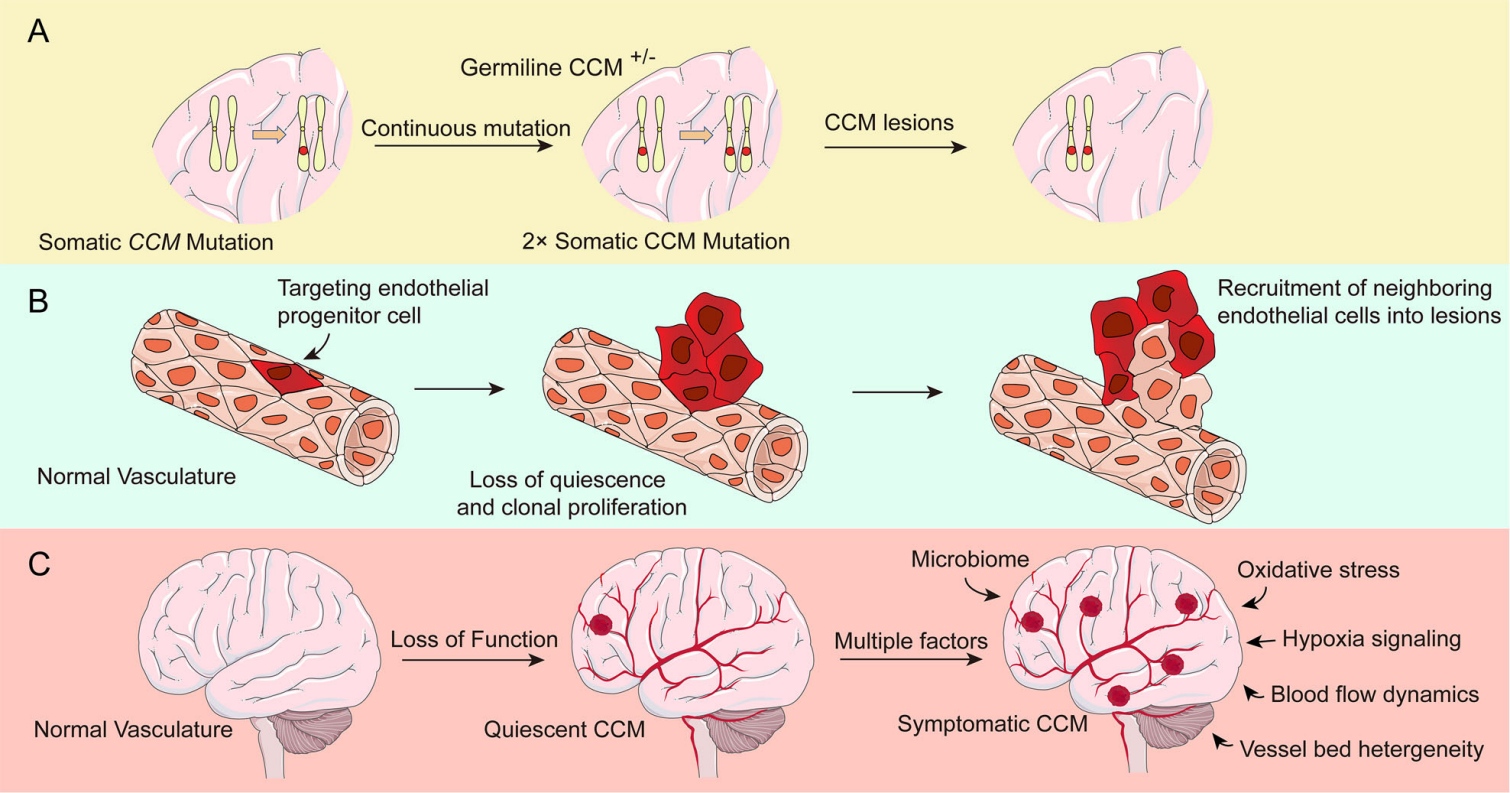
Mechanisms underlying the progression of CCM. **A** Genetic mutations contribute to the exacerbation of CCM formation. Germline mutations in CCM-related genes (e.g., CCM1, CCM2, CCM3) predispose individuals to lesion development. A second somatic mutation in endothelial cells results in biallelic loss, further aggravating CCM lesion formation. **B** Endothelial dysfunction and clonal expansion drive lesion growth. Normal vasculature undergoes pathological transformation as endothelial progenitor cells lose quiescence and proliferate clonally. The recruitment of neighboring endothelial cells further exacerbates lesion formation. **C** Transition from quiescent to symptomatic CCMs. Various systemic and microenvironmental factors, including microbiome alterations, oxidative stress, hypoxia signaling, blood flow dynamics, and vessel bed heterogeneity, contribute to lesion progression and symptomatic manifestations.

In contrast, familial CCMs are largely driven by inherited loss-of-function mutations in one of three core genes, CCM1 (KRIT1), CCM2 (Malcavernin), and CCM3 (PDCD10) **(**Fig. 1**)**, and show increased prevalence in Hispanic and Mexican-American populations, particularly in the southwestern United States [30–32]. When mutated, these genes cause structural and functional abnormalities in endothelial cells [33]. Disruption of CCM1 or CCM2 compromises tight and adherens junctions, resulting in impaired endothelial barrier function, aberrant vessel dilation, and progressive vascular remodeling that culminates in cavernous lesion formation [34, 35]. Mutations in CCM3 further exacerbate CCM progression, leading to more severe vascular abnormalities [36, 37].

Somatic activating mutations in PIK3CA represent a major driver in sporadic and aggressive CCM [38]. PIK3CA gain-of-function mutations often co-occur with loss-of-function mutations in CCM complex genes within the same endothelial cells, leading to marked upregulation of the PI3K–mTORC1 signaling axis and driving lesion growth through oncogenic-like mechanisms [25]. In mouse models, both endothelial CCM loss and PIK3CA activation are required for cavernoma formation, and pharmacologic inhibition of mTORC1 significantly suppresses lesion development, highlighting the functional contribution of PI3K downstream effectors [39]. Clinical sequencing studies further show a high prevalence of PIK3CA somatic mutations in human CCM lesions, with mutation status correlating with distinct clinicoradiological features, including increased brainstem involvement [25, 40]. Collectively, these findings indicate that PI3K signaling not only permits endothelial proliferation but also actively contributes to the pathological vascular remodeling in aggressive CCMs.

**Fig. 2:**
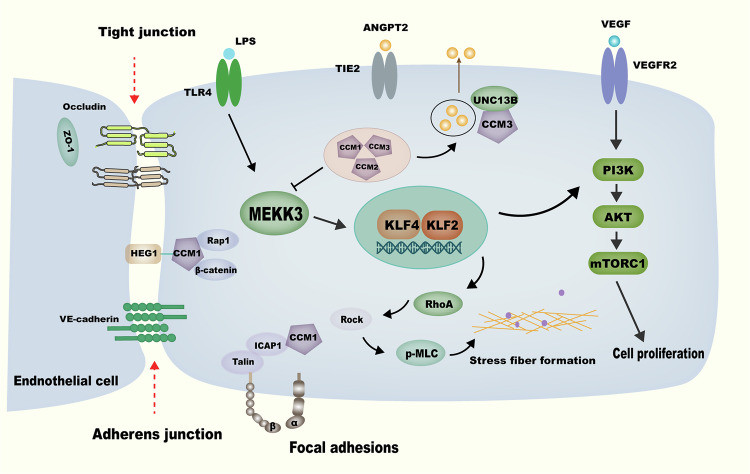
Schematic representation of MEKK3-mediated signaling pathways in CCM pathogenesis. This schematic illustrates the molecular interactions involved in endothelial dysfunction and CCM pathogenesis. The CCM heterotrimeric complex, consisting of CCM1 (KRIT1), CCM2, and CCM3 (PDCD10), functions as a negative regulator of MEKK3 signaling. Upstream inputs, including TLR4-mediated inflammatory signals and ANGPT2/TIE2 signaling, activate MEKK3, leading to the transcriptional upregulation of KLF2 and KLF4. These transcription factors regulate endothelial cell function through both PI3K-AKT-mTORC1 signaling, which promotes cell proliferation, and RHOA-ROCK-pMLC signaling, which drives cytoskeletal remodeling and stress fiber formation. In addition to these intracellular pathways, VEGFR2 signaling is depicted as a distinct input regulating endothelial proliferation. The CCM complex is also associated with HEG1, which stabilizes endothelial cell junctions through interactions with β-catenin and Rap1. Loss of CCM function results in dysregulated endothelial integrity, aberrant cytoskeletal dynamics, and excessive vascular permeability, all contributing to CCM lesion progression. These pathways collectively contribute to endothelial dysfunction and CCM lesion progression. The above-described mechanisms represent only a subset of well-characterized pathways that exacerbate CCM formation, and further research is required to elucidate additional contributing factors.

In parallel, MEKK3 (MAP3K3) has emerged as a pivotal integrative signaling node in CCM pathogenesis (Fig. 2) [41–43]. Recurrent somatic mutations in *MAP3K3*, notably the p.Ile441Met variant, are enriched in endothelial cells of sporadic CCM lesions and define a molecular CCM subclass with distinct imaging features [44]. Functional studies demonstrate that targeted expression of MAP3K3^I441M^ in brain endothelial cells using an rAAV-miniBEND vector is sufficient to induce cavernous malformations in mouse brains, highlighting its direct contribution to lesion formation [39, 45]. Beyond genetic alterations, MEKK3 is also known to respond to multiple environmental and inflammatory cues: for example, it mediates signaling downstream of IL-1R, TLR4 and IL-6 in response to Lipopolysaccharide (LPS) or pro-inflammatory cytokines, and can be activated by hemodynamic forces such as shear stress via mechanosensitive upstream regulators [46–48]. Together, these examples support the role of MEKK3 as a central integrator of diverse upstream signals, coordinating endothelial responses that shape vascular architecture and contribute to CCM lesion progression.

Collectively, these findings support a model in which PIK3CA and MEKK3 operate at distinct but interconnected levels, with MEKK3 appearing to play a more central role in coordinating downstream endothelial responses, rather than acting solely as a linear downstream effector of PI3K signaling [45].

## MEKK3 shapes the course of CCM progression

The progression of CCM involves the disruption of multiple endothelial signaling pathways that collectively compromise vascular stability. Imbalances in ANGPT2–TIE2 signaling weaken vascular integrity, increasing capillary permeability and promoting the formation of leakage-prone lesions [49]. Similarly, aberrant activation of VEGF–VEGFR2 signaling drives excessive angiogenic responses and endothelial proliferation. Mechanistically, VEGFR2 activation engages downstream PI3K–AKT signaling, which overlaps with and can be further amplified by somatic activating mutations in PIK3CA, promoting endothelial cell survival, proliferation, and clonal expansion [50–52]. While this pathway provides a permissive growth advantage, it alone cannot fully account for the complex vascular architecture and inflammatory activation characteristic of CCM lesions.

In parallel, chronic inflammatory signaling, particularly mediated by the LPS–TLR4 axis, contributes to CCM progression. LPS, largely derived from gut microbiota dysbiosis, activates TLR4-dependent inflammatory cascades, leading to the activation of NF-κB and MAPK pathways and the subsequent release of pro-inflammatory cytokines, including IL-6, TNF-α, and IL-1β [46, 53]. Given that LPS is largely derived from gut microbiota dysbiosis, these findings raise the possibility that microbiota-derived inflammatory signals may contribute to CCM pathogenesis.

Within this complex signaling landscape, MEKK3 has emerged as a pivotal integrative node [54, 55]. MEKK3 signaling plays a central role in maintaining endothelial barrier function, primarily through regulation of the KLF2/4 transcriptional cascade [2, 25, 56]. Aberrant MEKK3 activation leads to endothelial junctional instability and increased vascular permeability, thereby facilitating vascular malformation development [55, 57]. Importantly, MEKK3 integrates upstream angiogenic, genetic, and inflammatory cues, including signals mediated by TLR4 and YAP/TAZ, suggesting that microbiota-derived inflammatory signals may converge on MEKK3 to modulate endothelial responses and vascular dysfunction [14, 46, 58].

Although direct evidence is limited, gut microbiota and their metabolites may modulate MEKK3 activity in endothelial cells, potentially affecting vascular integrity and lesion progression in CCM.

## Gut-derived signals regulating brain MEKK3 activation in CCM progression

GBA is an integrated network involving the central nervous system (CNS), autonomic and enteric nervous systems, immune components, and endocrine signaling [59–61]. It enables bidirectional communication between the gut and brain through multiple routes, including the vagus nerve [62], microbial metabolites [63], cytokines [64], and neurotransmitters such as serotonin (5-HT) [65–67].

Among gut-derived signals, LPS, a microbial product of Gram-negative bacteria, has been widely implicated in cerebrovascular inflammation [68, 69]. In CCM, gut dysbiosis is associated with elevated circulating LPS levels, which activate inflammatory signaling in brain endothelial cells, prominently involving TLR4-dependent pathways [70, 71]. This activation contributes to endothelial barrier breakdown, leukocyte infiltration, and lesion progression [14]. Notably, MEKK3 functions as a key kinase downstream of TLR4, translating microbial signals into NF-κB-driven inflammatory cascades and tight junction disruption [72, 73].

**Fig. 3:**
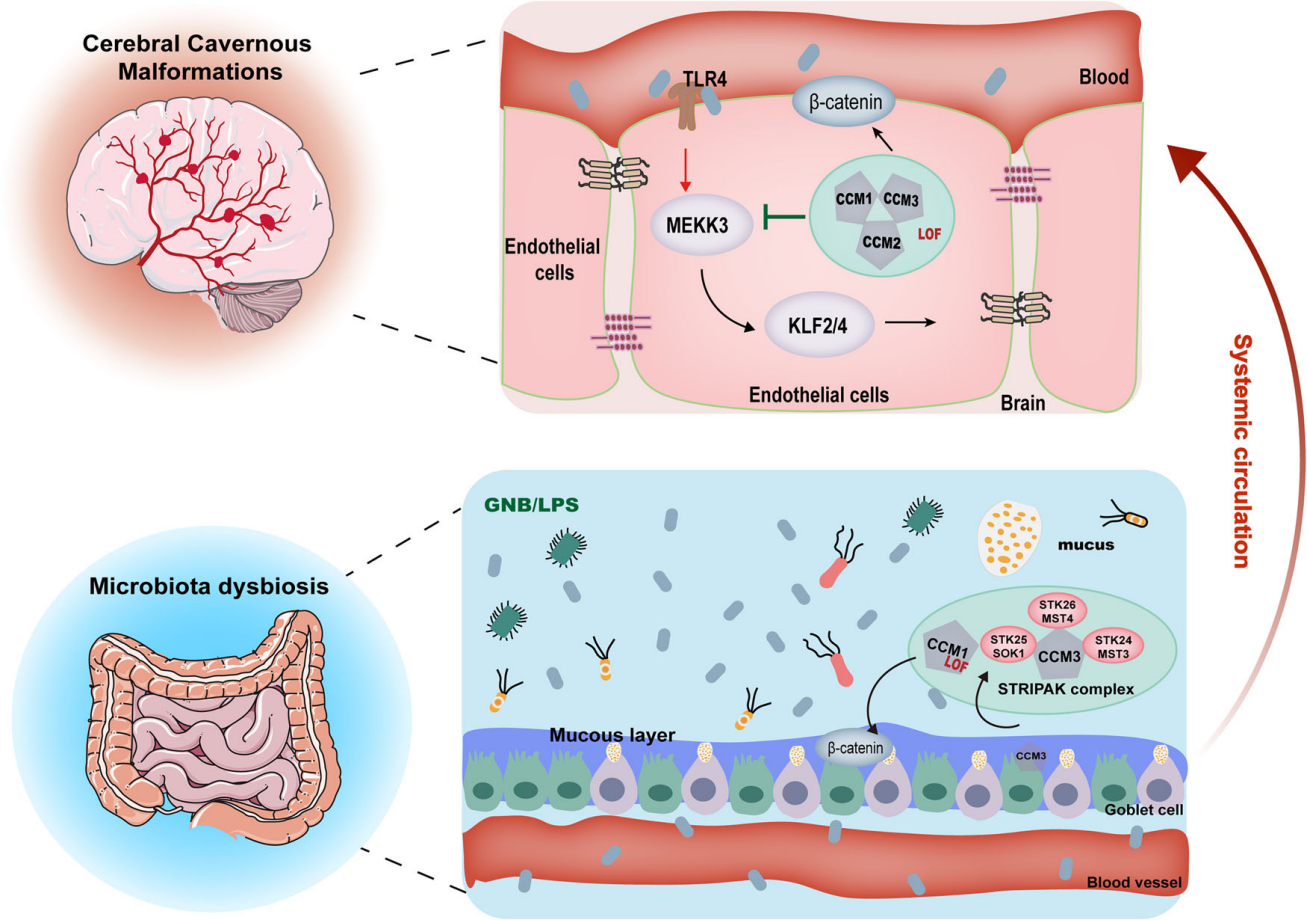
The gut-brain axis in CCM pathogenesis: the interplay between microbiota dysbiosis and endothelial dysfunction. Shown is a schematic representation of how microbiota dysbiosis contributes to CCM formation via systemic circulation. In the gut, Gram-negative bacteria-derived LPS disrupts the intestinal mucosal barrier, leading to increased permeability and translocation of bacterial components into the bloodstream. Loss-of-function mutations in the CCM complex (CCM1, CCM2, CCM3) disrupt the STRIPAK complex, affecting endothelial integrity and barrier function. Upon entering systemic circulation, LPS activates TLR4 signaling in brain endothelial cells, leading to aberrant MEKK3 activation and subsequent upregulation of KLF2/4 transcription factors. These molecular events compromise endothelial junctions and promote CCM lesion formation. Loss of CCM function further exacerbates β-catenin dysregulation, impairing vascular stability and contributing to disease progression. This diagram highlights the currently recognized mechanisms linking gut microbiota alterations to CCM lesion development via systemic inflammation and endothelial dysfunction. However, additional pathways may be involved, warranting further investigation.

In CCM patients, enrichment of LPS-producing bacteria, including *Streptococcus pyogenes* and *Campylobacter fetus*, has been reported, correlating with increased systemic inflammatory tone and cerebrovascular dysfunction (Fig. 3) [46, 74, 75]. Accordingly, modulation of the gut–brain axis via restoration of microbial balance, reduction of systemic endotoxemia, or pharmacological targeting of TLR4- and MEKK3-associated inflammatory signaling pathways represents a potential therapeutic strategy for cerebral cavernous malformations [76]. While specific MEKK3-targeted interventions are still in preclinical development, upstream modulatory strategies—including TLR4 antagonism and microbiota-based approaches designed to reduce LPS burden—merit further investigation as promising approaches to attenuate gut-to-brain inflammatory signaling in cerebral cavernous malformations [77, 78].

## Gut metabolites connecting the gut-brain axis and MEKK3 signaling in CCM

### Role of metabolites in CCM pathogenesis

#### SCFAs

SCFAs, including acetate, propionate, and butyrate, are major metabolic products derived from gut microbial fermentation of dietary fiber [79, 80]. For example, in both murine models and human CCM patients, alterations in gut microbiota have been reported, including an increased abundance of Gram-negative species such as *Odoribacter splanchnicus* and a decreased abundance of beneficial SCFA-producing taxa, including *Faecalibacterium prausnitzii*, which are associated with inflammation and endothelial dysfunction [81]. Some SCFAs enter systemic circulation and cross the blood-brain barrier (BBB), exerting regulatory effects on neuroinflammation and vascular homeostasis [82, 83].

The TLR4 signaling pathway has been identified as a key upstream regulator of MEKK3, with TLR4 activation enhancing MEKK3 expression and exacerbating pro-inflammatory signaling, thereby accelerating CCM pathogenesis. As a histone deacetylase (HDAC) inhibitor, butyrate modulates chromatin structure and suppresses TLR4 transcription at the epigenetic level [84, 85]. Additionally, it can indirectly attenuate TLR4-mediated inflammatory signaling by activating the GPR109A receptor [86–88]. Given the essential role of TLR4 in regulating MEKK3, butyrate-mediated TLR4 suppression may influence MEKK3 signaling [88]. Although this mechanism has yet to be directly validated, it opens new avenues for investigating the regulatory role of butyrate in CCM.

#### TMAO

TMAO, a gut microbiota-derived metabolite from dietary choline and carnitine, has been implicated in various cardiovascular diseases, including atherosclerosis and vascular dysfunction [89]. Gut bacteria capable of producing trimethylamine (TMA) from dietary choline and carnitine include *Escherichia coli* and *Desulfovibrio desulfuricans*, which contribute to systemic TMAO formation [90]. Elevated TMAO has been linked to endothelial dysfunction and vascular inflammation in cardiovascular disease [91], suggesting that dysbiosis of such TMA-producing bacteria may create a pro-inflammatory environment that could influence endothelial signaling and potentially modulate CCM lesion progression [92].

Emerging evidence suggests that TMAO can promote gut microbiota dysbiosis, alter lipid metabolism, and enhance systemic inflammation, all of which may potentially contribute to CCM progression [93, 94]. These inflammatory changes may subsequently enhance TLR4 expression and activate downstream signaling cascades, potentially leading to MEKK3 upregulation in CCM progression [95]. Investigating TMAO within the gut-brain axis framework could provide new perspectives on its role in CCM pathophysiology and potential therapeutic interventions.

#### LPS

Current research on the TLR4-MEKK3 signaling axis predominantly focuses on the role of LPS. LPS is produced by the outer membranes of gut Gram-negative bacteria such as *Bacteroides* species (e.g., *Bacteroides uniformis*, *Bacteroides vulgatus*) and Proteobacteria, including *Escherichia coli* and *Desulfovibrio* [78, 96, 97]. These bacteria are abundant sources of endogenous endotoxin in the gut and contribute to systemic LPS pools that can activate TLR4 and downstream signaling cascades critical to CCM pathogenesis [46, 53, 81, 98]. Upon binding TLR4, LPS forms a complex with co-receptors such as MD-2 and CD14, activating both MyD88-dependent and TRIF-dependent pathways, and induces the release of pro-inflammatory cytokines (TNF-α, IL-6, IL-1β) [99, 100].

As a key upstream regulator of MAP3K signaling cascades, persistent activation of the TLR4-MEKK3 axis disrupts endothelial cell adhesion by degrading VE-cadherin, increasing BBB permeability, and exacerbating CCM lesion progression [46]. Furthermore, LPS-induced MEKK3 signaling may enhance pro-inflammatory cytokines related to Th17 and Tc-17 phenotypes in symptomatic CCM patients, leading to vascular dilation and structural instability within CCM lesions [53]. Given the critical role of the LPS-TLR4-MEKK3 axis in CCM progression, targeted interventions such as TLR4 antagonists (e.g., TAK-242) and MEKK3 inhibitors hold promise as potential therapeutic strategies.

#### Indole and its derivatives

Indole is a major tryptophan metabolite produced by gut microbiota and plays a crucial role in gut-brain axis regulation [101]. Certain Gram-negative bacteria, such as *Escherichia coli* and *Bacteroides* species, utilize tryptophanase to degrade dietary tryptophan into indole and its derivatives, including indolepropionic acid (IPA), indoleacetic acid (IAA), and indolelactic acid (ILA) [102–104].

Indoxyl sulfate (IS), a key indole derivative, has been shown to activate TLR4, upregulating its expression and amplifying inflammatory signaling [105, 106]. Additionally, IS may induce reactive oxygen species (ROS) accumulation, which may further amplify MEKK3 phosphorylation and vascular injury [107, 108].

Conversely, certain indole metabolites, such as IPA, may exert protective effects against the TLR4 [109]. IPA, an aryl hydrocarbon receptor (AhR) agonist, has been reported to suppress TLR4 expression through its antioxidative and anti-inflammatory properties [110].

These findings suggest that dysregulated indole metabolism may modulate CCM progression through the TLR4-MEKK3 axis. Pro-inflammatory indole derivatives (e.g., IS) may accelerate CCM lesion development by promoting TLR4 signaling, MEKK3 activation, and vascular remodeling, while anti-inflammatory metabolites (e.g., IPA) may confer protective effects by suppressing the TLR4-MEKK3 pathway. Therefore, strategies aimed at restoring indole metabolic balance, such as reducing IS accumulation or increasing IPA levels, may represent novel approaches for CCM intervention.

### Immune cell involvement in CCM

#### Neutrophils

Immune cells, particularly neutrophils, may regulate MEKK3 signaling through multiple mechanisms, exacerbating endothelial damage and inflammation in CCM [111]. For example, *Escherichia coli* and *Bacteroides fragilis* can stimulate NET formation through LPS- or metabolite-mediated signaling, potentially enhancing TLR4-MEKK3 activation in brain endothelial cells [112, 113].

NET-associated histone H3 and DNA fragments are recognized by TLR4, upregulating MEKK3 expression [114, 115]. Furthermore, in inflammatory environments, neutrophils release elastase, myeloperoxidase (MPO), and pro-inflammatory cytokines (IL-1β, TNF-α), which not only compromise BBB integrity but may also enhance MEKK3 signaling via the TLR4/NF-κB axis, driving endothelial proliferation, migration, and vascular remodeling [116–118].

#### Macrophages

Additionally, M1 macrophages amplify pro-inflammatory signaling through the TLR4, while M2 macrophages may counteract this by secreting IL-10 and TGF-β, potentially inhibiting MEKK3 activity [119]. Th17 cells secrete IL-17, which may indirectly enhance MEKK3 activation, whereas Treg cells may suppress NF-κB signaling and reduce MEKK3 expression [120]. Notably, gut microbial constituents and their metabolites can influence this balance of immune responses. For example, enteric Proteobacteria such as *Escherichia coli* and members of the *Enterobacteriaceae* can skew macrophage polarization toward the M1 phenotype through LPS-TLR4 priming [121–124]. These findings suggest that CCM progression is regulated by both direct MEKK3 activation and immune cell-mediated modulation. Targeting neutrophil-mediated mechanisms, such as NET inhibition or chemokine blockade, may serve as novel strategies to regulate MEKK3 signaling and slow CCM progression.

The gut-brain axis orchestrates multiple regulatory layers influencing the TLR4-MEKK3 pathway in CCM progression, integrating microbial metabolites and immune cell interactions. However, current research primarily focuses on TLR4-dependent mechanisms, while other potential pathways modulating MEKK3 remain underexplored. Future investigations should delve into alternative gut-brain axis-mediated mechanisms of MEKK3 regulation, broadening therapeutic avenues for CCM. A summary of relevant studies is presented in Table 1.

**Table 1:** Immune Cells and Metabolites in MEKK3 Pathway Regulation.

Metabolites / Immune Cells	Effects	Mechanism	References
SCFAs	1. LPS/TLR4 2. Regulation of blood-brain barrier 3. Production of SCFAs	1. Systemic Circulation 2. Neurovascular Circulation	[80, 81, 83–88]
LPS	1. LPS/TLR4 2. Promotion of inflammatory cytokine	1. Systemic Circulation 2. Lymphatic Circulation 3. Neurovascular Circulation	[47, 54, 79, 82, 97–101]
Indoles and their derivatives	1. LPS/TLR4 2. Neuroprotective 3. Regulation of blood-brain barrier 4. Promotion of inflammatory cytokine	1. Systemic Circulation 2. Neurovascular Circulation	[105–110]
Neutrophils	1. LPS/TLR4 2. Promotion of inflammatory cytokine	1. Systemic Circulation 2. Cerebrospinal Fluid Circulation	[114–118]
Macrophages	1. LPS/TLR4 2. Neuroprotective 3. Regulation of blood-brain barrier 4. Promotion of inflammatory cytokine	1. Systemic Circulation 2. Lymphatic Circulation 3. Neuroimmune Circulation	[119, 121–124]
Th17/Treg Cells	1. LPS/TLR4 3. Regulation of blood-brain barrier	1. Systemic Circulation 2. Lymphatic Circulation 3. Neuroimmune Circulation	[120]

## Conclusion

CCM is a complex and potentially life-threatening cerebrovascular disease that presents significant challenges in pathology and treatment [125]. Although scientific advancements have provided some insights, many questions regarding its pathogenesis and therapeutic strategies remain unresolved. Against this backdrop, growing attention has been directed toward the gut-brain axis, emerging as a promising avenue in CCM research. The gut microbiota and its metabolites play a crucial role in maintaining immune homeostasis and vascular health, and their interactions with the cerebrovascular system may profoundly influence CCM onset and progression.

The potential link between the gut-brain axis and CCM offers a novel research perspective [14]. Dysbiosis of the gut microbiota and alterations in its metabolites, such as SCFAs and TMAO, are closely associated with endothelial function, BBB integrity, and inflammatory responses, potentially driving CCM pathogenesis. For instance, SCFAs enhance BBB integrity by modulating tight junction protein expression in endothelial cells, whereas TMAO may exacerbate vascular dysfunction and promote CCM progression by activating inflammatory pathways [94, 126]. These findings suggest that targeting the gut-brain axis could be a viable strategy for controlling and mitigating CCM progression.

While these findings support the gut-brain axis as a promising therapeutic target for CCM, a key unanswered question lies in the intracellular signaling mechanisms that mediate this gut-to-brain communication. In this context, MEKK3emerges as a compelling candidate. Although current research into MEKK3 within the gut-brain axis remains limited and has been primarily linked to responses to endotoxins such as LPS, its well-characterized role in cerebral cavernous malformations, particularly in mediating endothelial stress responses and vascular malformation formation, justifies further investigation into its broader systemic effects. Given its upstream localization in key inflammatory and barrier-regulatory pathways, MEKK3 may act as a molecular hub that integrates microbial signals from the gut to modulate cerebrovascular pathophysiological outcomes.

Therefore, targeting MEKK3 may offer a novel and mechanistically grounded approach to modulating gut-brain axis dysfunction in CCM. Future therapeutic strategies might combine modulation of gut microbiota (e.g., via probiotics, prebiotics, or dietary changes to enhance SCFAs and reduce TMAO) with inhibition of MEKK3-related signaling cascades to restore vascular integrity and suppress lesion progression. However, the clinical relevance and mechanistic precision of such interventions remain to be rigorously tested. Further studies are needed to clarify how gut-derived signals interface with MEKK3 activity and to determine whether this axis can be therapeutically leveraged to slow or prevent CCM progression.
